# Severe obesity and the impact of medical weight loss on estimated glomerular filtration rate

**DOI:** 10.1371/journal.pone.0228984

**Published:** 2020-02-25

**Authors:** Amy E. Rothberg, Laura N. McEwen, William H. Herman

**Affiliations:** 1 Department of Internal Medicine, University of Michigan, Ann Arbor, Michigan, United States of America; 2 Department of Nutritional Sciences, University of Michigan, Ann Arbor, Michigan, United States of America; 3 Department of Epidemiology, University of Michigan, Ann Arbor, Michigan, United States of America; Weill Cornell Medical College Qatar, QATAR

## Abstract

**Objective:**

To assess the impact of obesity, glucose tolerance, and weight loss on renal function, we measured serum creatinine and cystatin C and estimated glomerular filtration rate (GFR) indexed to 1.73m^2^ body surface area (BSA) and GFR indexed to actual BSA in subjects with normal and abnormal glucose tolerance before and up to 2 years after medical weight loss.

**Methods:**

We studied 146 subjects at baseline and 3-to-6 months after 18% reduction in weight; 43 were also studied at 2-years. GFR was estimated using the MDRD, CKD-EPI_Cr_, CKD-EPI_CysCr_, and the CKD-EPI_Cys_ equations.

**Results:**

eGFR was consistently lower when creatinine-based rather than cystatin C-based estimating equations were used. eGFR was lower when creatinine-based or cystatin C-based equations were indexed to 1.73m^2^ BSA than when they were indexed to actual BSA. eGFR indexed to actual BSA was more likely to demonstrate hyperfiltration (eGFR ≥135 ml/min) than eGFR indexed to 1.73m^2^ BSA and decreased into the normal range with weight loss. eGFR was highest in subjects with impaired fasting glucose but there was little difference in the patterns of change in eGFR across groups by glucose tolerance status.

**Conclusions:**

With severe obesity, high fat-free mass and BSA result in low estimates of eGFR indexed to 1.73m^2^ BSA, especially when creatinine-based estimating equations are used. GFR indexed to actual BSA is approximately 50% higher. When eGFR is indexed to actual BSA, many subjects display evidence of renal hyperfiltration which improves with weight loss. In subjects with severe obesity undergoing medical weight loss, estimating equations that use cystatin C and are indexed to actual BSA may provide a more accurate assessment of renal function.

## Introduction

Numerous studies have demonstrated an association between obesity and chronic kidney disease (CKD) [1]. Higher body mass index (BMI) has been associated with lower estimated glomerular filtration rate (eGFR), loss of eGFR over time, and incident end-stage renal disease (ESRD). The mechanisms by which obesity causes or worsens CKD remain unclear. Some of the deleterious effects of obesity are mediated by comorbid conditions including hypertension and diabetes. Obesity may also impact the kidneys directly by its effects on adipokines, inflammation, oxidative stress, activation of the renin-angiotensin-aldosterone system, sympathetic activation, insulin resistance, and abnormal lipid metabolism [1].

A clinical trial demonstrated that calorie restriction and increased physical activity can reduce the incidence of CKD by 30% in patients with type 2 diabetes [2], and systematic reviews have demonstrated that interventions to reduce body weight may reduce blood pressure, proteinuria, and glomerular hyperfiltration [3,4]. Based upon this evidence, international associations focused on kidney disease and Healthy People 2020 have recommended interventions to reduce body weight in obese people at risk for CKD and in those with early CKD, especially those with hypertension and diabetes.

A limitation to many clinical trials and virtually all population-based prospective observational studies is that glomerular filtration rate (GFR) was assessed indirectly using either the Modification of Diet in Renal Disease (MDRD) equation or the Chronic Kidney Disease Epidemiology Collaboration (CKD-EPI_Cr_) equation that estimate GFR from serum creatinine and age, sex, and race [5,6]. It has been recognized that creatinine-based estimating equations may be inaccurate in people with extremes of muscle mass including those with severe obesity. Obesity is associated with increased fat-free mass and individuals with higher fat-free mass have higher serum creatinine levels and lower GFR as estimated by the MDRD and CKD-EPI_Cr_ equations than individuals with lower fat-free mass [7]. For this reason, cystatin C has been suggested as an alternative for estimating GFR in people with high fat-free mass [8]. Although some studies have found an association between cystatin C and BMI [9, 10], percent body fat [11], and diabetes [10], the CKD-EPI cystatin C equation (CKD-EPI_Cys_) has been shown to have advantages over creatinine-based eGFR equations in patients in whom muscle mass is abnormally high [12]. More recently, another GFR estimating equation has been developed and validated based on cystatin C in combination with creatinine (CKD-EPI_CysCr_) [13]. It has been reported to perform better than equations based on either of these markers alone, and appears to be especially valuable in patients whose eGFR based on creatinine is 45–74 ml/min/1.73^2^ [14].

Routinely indexing eGFR to 1.73m^2^ body surface area (BSA) also complicates the assessment of eGFR in severely obese patients. Since the 1920s, GFR has been indexed routinely to 1.73m^2^ BSA, since at that time, the average American BSA at age 25 years was 1.73m^2^. Studies in rabbits and dogs had demonstrated an association between BSA, kidney weight, and urea excretion, and indexing GFR to BSA was recommended to reduce the variability in urea clearance and creatinine clearance between children and adults [15,16]. Although indexing GFR to BSA has very little impact on GFR in normal body size individuals, the consequences can be quite substantial in individuals with severe obesity and the appropriateness of adjusting to 1.73m^2^ BSA has been questioned in severely obese people.

Another issue in estimating GFR relates to the impact of substantial weight loss on serum creatinine and cystatin C and thus its impact on eGFR. A widely cited rule guiding expected loss of fat-free mass with weight loss states that approximately one-quarter of lost weight will be fat-free mass and the remaining three-quarters will be fat mass [17]. In reality, the proportion of weight lost as fat-free mass and fat mass varies over time and is influenced by age, sex, baseline adiposity, energy intake, dietary composition, and level and type of physical activity [17]. Nevertheless, any reduction in fat-free mass would be expected to reduce serum creatinine and increase eGFR. A recent study demonstrated that when patients experience large weight loss following bariatric surgery, muscle mass and serum creatinine are reduced and both the MDRD and CKD-EPI_Cr_ equations overestimate measured GFR indexed to 1.73m^2^ BSA [18]. In contrast, cystatin C-based eGFR indexed to 1.73m^2^ BSA is unchanged after weight loss [18].

The purpose of this study was to assess the association between severe obesity, serum creatinine, and cystatin C, and to assess the impact of substantial medical weight loss on eGFR in individuals with normal fasting glucose (NFG), impaired fasting glucose (IFG), and type 2 diabetes (T2DM) in the short-term (3-to-6 months) and long-term (2-years). We also sought to describe the impact of severe obesity and substantial medical weight loss on eGFR indexed to 1.73m^2^ BSA and indexed to actual BSA.

## Methods

We studied 146 patients (72 with NFG, 33 with IFG, and 41 with T2DM) enrolled in the University of Michigan Weight Management Program (WMP). The WMP is an intensive, behavioral weight management program that employs very low energy diet for three-to-six months to achieve 15% reduction in body weight, followed by reintroduction of regular food stuffs to maintain weight loss for a total of 2 years. For the first 3-to-6 months, participants are encouraged to engage in moderate physical activity such as brisk walking for 30 minutes per day 5 days per week. From 3-to-6 months to 2-years, participants are encouraged to engage in vigorous physical activity sufficient to cause breathlessness and sweating for 60 minutes per day 5 days per week. The research program was reviewed and approved by the Institutional Review Board of the University of Michigan and all participants provided written informed consent.

Subjects were included if they had assessments of sociodemographic characteristics (age, sex, race) and clinical parameters (height, weight, BMI, waist circumference, blood pressure, and body composition by DEXA) and if they had stored fasting serum and urine specimens obtained at baseline, at 3-to-6 months after weight loss, and at 2-years after enrollment if still enrolled. Subjects were excluded if they were missing any of these variables. BSA was calculated according to the methods of Livingston and Lee [19]. Glucose tolerance status at baseline was classified according to American Diabetes Association criteria [20].

Serum and urine specimens were aliquoted, stored at -70°C, and assayed for serum creatinine, serum cystatin C, urine creatinine, and urine albumin at the end of the study. Serum creatinine and urine creatinine were measured using a Randox RX Series Daytona chemistry analyzer. This assay is traceable to the National Institute of Standards and Technology Creatinine Standard Reference Materials 909b and 967. Serum cystatin C was measured with a particle enhanced immunoturbidimetric assay (Tina-quant Cystatin C Gen.2 (CYSC2)) using a Roche Cobas c 502 Analyzer. Urine albumin was measured with a Sekure Chemistry Microalbumin Assay Kit from Sekisui Diagnostics using a Roche Cobas Mira Chemistry Analyzer. For serum creatinine, the within run precision as assessed by the percent coefficient of variation (intraassay CV%) was 4.0% at a serum creatinine level of 0.75 mg/dl and 2.6% at a serum creatinine level of 1.5 mg/dl. For cystain C, the intraassay CV% was 4.4% at 0.95 mg/L and 3.7% at 1.90 mg/L. For urine creatinine, the intraassay CV% was 2.1% at 51 mg/dl and 2.1% at 102 mg/dl. For urine microalbumin, the intraassay CV% was 4.8% at 5.8 mg/l and 3.9% at 45 mg/l. All assays were performed by the Michigan Diabetes Research Center Chemistry Laboratory.

First, we described serum creatinine, cystatin C, and urine albumin-to-creatinine ratio (ACR). We then assessed GFR indexed to 1.73m^2^ BSA using each of the four estimating equations [5,6,12,13]. We also assessed eGFR indexed to actual BSA by multiplying indexed eGFR by BSA/1.73m^2^ [16]. We examined the normality of each variable using the Shapiro-Wilk test for normality. Since many of the variables were not normally distributed, we used the Wilcoxon (nonparametric) test to assess the difference in variables between baseline and 3-to-6 months or 2-years and between 3-to-6 months and 2-years. We used generalized linear models to assess differences in the population by glucose tolerance status.

## Results

Table 1 shows the characteristics of the 146 subjects studied at baseline and again at 3-to-6 months after weight loss. Mean age was 50 years, 48% of subjects were men, and 94% were white. Median BMI was 39 kg/m^2^ and median weight was 117 kg. Median BSA at baseline was 2.54 m^2^. Median serum creatinine was 0.95 mg/dl and cystatin C was 0.83 mg/L. Median urine albumin-to-creatinine ratio was 3.9 mg/g with an interquartile range (IQR) of 2.5 to 6.0 mg/g. At baseline, only 2 of 146 subjects had urine albumin-to-creatinine ratios ≥30 mg/g.

**Table 1:** Characteristics of the total population studied at baseline and 3-to-6 months. Data are presented as N(%), mean ± standard deviation, or median (interquartile range).

	Total Population		Glucose Tolerance Status					
Characteristic	N = 146		NFG N = 72		IFG N = 33		T2DM N = 41	
Age (years)	50 ± 9		48 ± 10		50 ± 7		54 ± 7	
Sex (% male)	48%		42%		58%		51%	
Race (% white)	94%		93%		94%		95%	
	**Baseline**	**3-to-6 Mos**	**Baseline**	**3-to-6 Mos**	**Baseline**	**3-to-6 Mos**	**Baseline**	**3-to-6 Mos**
BMI (kg/m^2^)	39 (36–43)	32 (30–36)	39 (36–42)	32 (30–35)§	43 (38–48)	33 (31–39)§	39 (36–42)	33 (30–35)§
Weight (kg)	117 (105–132)	96 (86–106)§	116 (104–127)	95 (83–104)§	127 (109–148)	103 (90–122)‡	115 (105–130)	97 (90–104)§
BSA (m^2^)	2.54 (2.38–2.75)	2.24 (2.09–2.39)§	2.53 (2.36–2.69)	2.23 (2.04–2.37)§	2.68 (2.43–2.97)	2.35 (2.15–2.62)‡	2.51 (2.38–2.73)	2.26 (2.16–2.37)§
Waist circumference (cm)	120 (110–130)	102 (96–112)§	115 (109–124)	98 (94–106)§	126 (115–138)	111 (98–118)§	120 (115–133)	105 (99–115)§
Fat-free mass (kg)	62 (52–72)	58 (49–69)*	59 (50–69)	56 (47–66)	65 (54–72)	65 (53–71)	65 (56–79)	58 (53–71)
Fat mass (kg)	51 (44–62)	36 (28–44)§	51 (46–62)	35 (28–45)§	55 (44–68)	40 (28–48)†	49 (44–59)	35 (28–41)§
Systolic BP (mmHg)	132 (120–139)	121 (113–131)§	129 (119–138)	123 (115–133)*	136 (127–148)	123 (115–130)†	132 (121–138)	120 (112–129)‡
Serum Creatinine (mg/dl)	0.95 (0.87–1.05)	0.91 (0.85–1.00)*	0.95 (0.87–1.05)	0.91 (0.85–1.0)	0.93 (0.89–1.05)	0.91 (0.88–0.99)	0.96 (0.89–1.08)	0.93 (0.84–1.00)
Cystatin C (mg/L)	0.83 (0.74–0.90)	0.78 (0.72–0.88)	0.81 (0.72–0.88)	0.76 (0.69–0.85)	0.84 (0.75–0.91)	0.80 (0.74–0.87)	0.84 (0.76–0.98)	0.85 (0.76–0.98)
U albumin-to-creatinine (mg/g)	3.9 (2.5–6.0)	4.5 (2.8–7.4)	3.6 (2.3–5.9)	4.1 (2.7–7.0)	3.7 (2.5–5.5)	4.9 (3.0–6.9)	4.5 (2.7–6.2)	4.7 (3.1–7.6)
UACR ≥30 (mg/g)||	N = 2 (1%)	N = 1 (1%)	N = 1 (1%)	N = 0 (0%)	N = 0 (0%)	N = 0 (0%)	N = 1 (2%)	N = 1 (2%)

*p<0.05

†p<0.01

‡p<0.001

§p<0.0001 compared to baseline

|| cell size too small to do statistical testing

At baseline, serum creatinine was significantly and positively associated with male sex and fat free mass and negatively associated with BMI and fat mass. It was not associated with age, race, waist circumference, or glucose tolerance. Cystatin C was positively associated with age, male sex, waist circumference, and type 2 diabetes. It was not associated with race, BMI, fat free mass, or fat mass. In multivariate models, serum creatinine at baseline was associated with male sex and cystatin C with age.

After three-to-six months of very low energy diet followed by a one month period of weight stabilization with normal foodstuff, median weight decreased by 21 kg (18%), BMI decreased by 7 kg/m^2^, and BSA decreased by 0.30 m^2^. Weight loss was associated with a 4 kg reduction in fat-free mass and a 15 kg reduction in fat mass. Serum creatinine decreased from 0.95 to 0.91 mg/dl and cystatin C from 0.83 to 0.78 mg/L (Table 1).

In a multivariate model incorporating baseline values of serum creatinine, BMI, waist circumference, fat free mass, and fat mass and change in BMI, waist circumference, fat free mass, and fat mass at 3-to-6 months, change in serum creatinine at 3-to-6 months was negatively associated with serum creatinine and BMI at baseline and positively associated with fat free mass and fat mass at baseline and change in fat mass at 3-to-6 months. In a similar multivariate model in which cystatin C was substituted for serum creatinine, change in cystatin C was negatively associated with cystatin C at baseline. No other variables were associated with change in cystatin C at 3-to-6 months.

Table 1 also shows the baseline characteristics of the subjects with NFG, IFG, and T2DM. Subjects with T2DM were significantly older than those with NFG (p<0.05). There was no difference in sex or race between the groups. Baseline BMI was higher in subjects with IFG than in those with NFG (p<0.05). Median baseline weight, BSA, and waist circumference were greatest in subjects with IFG. Fat-free mass and fat mass did not differ among the groups with NFG, IFG, or T2DM. Both serum creatinine and cystatin C levels tended to be higher in subjects with T2DM. Only the difference in cystatin C levels between subjects with T2DM and NFG were statistically significant (p<0.05).

The decrease in BMI and weight was greatest in subjects with IFG. The decrease in BSA, waist circumference, fat-free mass, and fat mass did not differ across the groups by glucose tolerance status. Serum creatinine decreased by 0.03 mg/dL in subjects with T2DM, and 0.02 mg/dL in those with IFG, and 0.04 mg/dL in those with NFG (p = NS). Cystatin C changed only modestly in each glucose tolerance group (+0.01 mg/L in T2DM -0.04 mg/L in IFG and -0.05 mg/L in those with NFG) (p = NS).

Table 2 shows eGFR indexed to 1.73m^2^ BSA and to actual BSA at baseline using each of the four estimating equations. Table 2 also shows the distribution of eGFR levels according to thresholds of eGFR ≥135 (defined as hyperfiltration), 120–134, 90–119, 60–89, and <60 ml/min/1.73m^2^ or ml/min. In general, eGFR indexed to 1.73m^2^ BSA at baseline was lowest when creatinine-based estimating equations were used (lower with the MDRD than the CKD-EPI_Cr_ equation), intermediate when the CKD-EPI_CysCr_ equation was used, and highest when the CKD-EPI_Cys_ equation was used. When eGFR was indexed to 1.73m^2^ BSA, no participants had evidence of hyperfiltration and many participants had eGFR between 60 and 89 ml/min/1.73m^2^ (79%, 73%, 47%, and 25% respectively). The remaining participants had GFRs <60 ml/min/1.73m^2^ (13%, 5%, 3%, and 2% respectively) or between 90 and 119 ml/min/1.73m^2^ (8%, 23%, 49%, and 66% respectively). In every instance, eGFR indexed to 1.73m^2^ BSA was substantially lower than eGFR indexed to actual BSA. When eGFR was indexed to actual BSA (Table 2), mean eGFRs were approximately 50% higher. The mean eGFR values were 108, 119, 133, and 146 ml/min and 15%, 23%, 43%, and 61% of subjects had eGFR ≥135 ml/min when the MDRD, CKD-EPI_Cr_, CKD-EPI_CysCr_, and CKD-EPI_Cys_ equations were used.

**Table 2:** eGFR indexed to 1.73m^2^ BSA and to actual BSA at baseline and 3-to-6 months by estimating equation and glucose tolerance status. Data are presented as N(%) or mean ± standard deviation.

	Total Population		Glucose Tolerance Status					
	N = 146		NFG N = 72		IFG N = 33		T2DM N = 41	
eGFR Equation	Baseline	3-to-6 Mos	Baseline	3-to-6 Mos	Baseline	3-to-6 Mos	Baseline	3-to-6 Mos
MDRD/1.73m^2^	72 ± 12	76 ± 12§	73 ± 11	76 ± 11†	75 ± 11	78 ± 10	69 ± 13	74 ± 13§
≥135	0 (0%)	0 (0%)	0 (0%)	0 (0%)	0 (0%)	0 (0%)	0 (0%)	0 (0%)
120–134	0 (0%)	0 (0%)	0 (0%)	0 (0%)	0 (0%)	0 (0%)	0 (0%)	0 (0%)
90–119	12 (8%)	16 (11%)	7 (10%)	9 (13%)	4 (12%)	4 (12%)	1 (2%)	2 (7%)
60–89	115 (79%)	120 (82%)	56 (78%)	59 (82%)	28 (85%)	29 (88%)	31 (76%)	32 (78%)
<60	19 (13%)	10 (7%)	9 (13%)	4 (6%)	1 (3%)	0 (0%)	9 (22%)	6 (15%)
MDRD indexed to actual BSA	108 ± 25	99 ± 20§	107 ± 22	97 ± 19§	118 ± 29	106 ± 23‡	103 ± 23	97 ± 20‡
≥135	21 (15%)	6 (4%)	7 (10%)	2 (3%)	10 (30%)	3 (9%)	4 (10%)	1 (2%)
120–134	19 (13%)	20 (14%)	10 (14%)	10 (14%)	3 (9%)	7 (21%)	6 (15%)	3 (7%)
90–119	72 (49%)	70 (48%)	42 (58%)	33 (46%)	15 (45%)	14 (42%)	15 (37%)	23 (56%)
60–89	33 (23%)	47 (32%)	13 (18%)	26 (36%)	5 (15%)	9 (27%)	15 (37%)	12 (29%)
<60	1 (1%)	3 (2%)	0 (0%)	1 (1%)	0 (0%)	0 (0%)	1 (2%)	2 (5%)
CKD-EPI_Cr_ /1.73m^2^	80 ± 12	79 ± 12§	81 ± 12	81 ± 12§	82 ± 10	82 ± 10§	76 ± 14	76 ± 14§
≥135	0 (0%)	0 (0%)	0 (0%)	0 (0%)	0 (0%)	0 (0%)	0 (0%)	0 (0%)
120–134	0 (0%)	0 (0%)	0 (0%)	0 (0%)	0 (0%)	0 (0%)	0 (0%)	0 (0%)
90–119	33 (23%)	33 (23%)	17 (24%)	17 (24%)	9 (27%)	9 (27%)	7 (17%)	7 (17%)
60–89	106 (73%)	105 (72%)	52 (72%)	52 (72%)	24 (73%)	24 (73%)	30 (73%)	29 (71%)
<60	7 (5%)	8 (5%)	3 (4%)	3 (4%)	0 (0%)	0 (0%)	4 (10%)	5 (12%)
CKD-EPI_Cr_ indexed to actual BSA	119 ± 26	104 ± 22§	119 ± 24	103 ± 21§	129 ± 29	111 ± 24§	112 ± 25	99 ± 20§
≥135	34 (23%)	14 (10%)	16 (22%)	6 (8%)	11 (33%)	7 (21%)	7 (17%)	1 (2%)
120–134	27 (18%)	17 (12%)	12 (17%)	7 (10%)	5 (15%)	6 (18%)	10 (24%)	4 (10%)
90–119	66 (45%)	76 (52%)	35 (49%)	42 (58%)	14 (42%)	13 (39%)	17 (41%)	21 (51%)
60–89	18 (12%)	37 (25%)	9 (13%)	17 (24%)	3 (9%)	7 (21%)	6 (15%)	13 (32%)
<60	1 (1%)	2 (1%)	0 (0%)	0 (0%)	0 (0%)	0 (0%)	1 (2%)	2 (5%)
CKD-EPI_CysCr_/1.73m^2^	89 ± 14	93 ± 13§	92 ± 13	95 ± 12‡	91 ± 10	94 ± 9*	84 ± 16	87 ± 16*
≥135	0 (0%)	0 (0%)	0 (0%)	0 (0%)	0 (0%)	0 (0%)	0 (0%)	0 (0%)
120–134	1 (1%)	2 (1%)	1 (1%)	1 (1%)	0 (0%)	1 (3%)	0 (0%)	0 (0%)
90–119	72 (49%)	85 (58%)	38 (53%)	44 (61%)	18 (55%)	21 (64%)	16 (39%)	20 (49%)
60–89	69 (47%)	56 (38%)	33 (46%)	27 (38%)	15 (45%)	11 (33%)	21 (51%)	18 (44%)
<60	4 (3%)	3 (2%)	0 (0%)	0 (0%)	0 (0%)	0 (0%)	4 (10%)	3 (7%)
CKD-EPI_CysCr_ indexed to actual BSA	133 ± 27	121 ± 22§	134 ± 24	122 ± 21§	142 ± 28	128 ± 22§	125 ± 29	114 ± 22§
≥135	63 (43%)	38 (26%)	32 (44%)	18 (25%)	19 (58%)	12 (36%)	12 (29%)	8 (20%)
120–134	43 (29%)	33 (23%)	22 (31%)	19 (26%)	8 (24%)	7 (21%)	13 (32%)	7 (17%)
90–119	34 (23%)	68 (47%)	17 (24%)	34 (47%)	6 (18%)	13 (39%)	11 (27%)	21 (51%)
60–89	6 (4%)	7 (5%)	1 (1%)	1 (1%)	0 (0%)	1 (3%)	5 (12%)	5 (12%)
<60	0 (0%)	0 (0%)	0 (0%)	0 (0%)	0 (0%)	0 (0%)	0 (0%)	0 (0%)
CKD-EPI_Cys_/1.73m^2^	98 ± 16	100 ± 16†	101 ± 15	104 ± 14†	98 ± 13	101 ± 12*	92 ± 19	92 ± 19
≥135	0 (0%)	0 (0%)	0 (0%)	0 (0%)	0 (0%)	0 (0%)	0 (0%)	0 (0%)
120–134	10 (7%)	12 (8%)	8 (11%)	9 (13%)	1 (3%)	1 (3%)	1 (2%)	2 (5%)
90–119	96 (66%)	100 (68%)	48 (67%)	52 (72%)	24 (73%)	26 (78%)	24 (59%)	22 (54%)
60–89	37 (25%)	31 (21%)	16 (22%)	11 (15%)	8 (24%)	6 (18%)	13 (32)	14 (34%)
<60	3 (2%)	3 (2%)	0 (0%)	0 (0%)	0 (0%)	0 (0%)	3 (7%)	3 (7%)
CKD-EPI_Cys_ indexed to actual BSA	146 ± 29	131 ± 24§	148 ± 27	133 ± 23§	153 ± 28	137 ± 23§	136 ± 32	121 ± 25§
≥135	89 (61%)	61 (42%)	45 (63%)	33 (46%)	24 (73%)	17 (52%)	20 (49%)	11 (27%)
120–134	30 (21%)	34 (23%)	16 (22%)	15 (21%)	3 (9%)	9 (27%)	11 (27%)	10 (24%)
90–119	22 (15%)	44 (30%)	10 (14%)	22 (31%)	6 (18%)	6 (18%)	6 (15%)	16 (39%)
60–89	5 (3%)	7 (5%)	1 (1%)	2 (3%)	0 (0%)	1 (3%)	4 (10%)	4 (10%)
<60	0 (0%)	0 (0%)	0 (0%)	0 (0%)	0 (0%)	0 (0%)	0 (0%)	0 (0%)

*p<0.05

†p<0.01

‡p<0.001

§p<0.0001 compared to baseline

At 3-to-6 months following 18% weight loss, eGFR indexed to 1.73m^2^ BSA increased slightly except when the CKD-EPI_Cr_ equation was used in which case it decreased slightly (Table 2). When eGFR was indexed to actual BSA, eGFR decreased by approximately 10% at 3-to-6 months regardless of which equation was used. The percentage of subjects with eGFR ≥135 ml/min decreased when any of the estimating equations were used (Table 2).

Table 2 also shows eGFR at baseline and at 3-to-6 months by glucose tolerance status. Within groups, eGFR indexed to 1.73m^2^ BSA was lower with the MDRD equation compared to the CKD-EPI_Cr_ equation compared to the CKD-EPI_CysCr_ equation compared to the CDK-EPI_Cys_ equation. When the MDRD estimating equation indexed to 1.73m^2^ was used, GFR tended to increase with weight loss in each of the groups by glucose tolerance status. When the CKD-EPI_Cr_ estimating equation was used, GFR indexed to 1.73m^2^ BSA decreased overall and in each of the glucose tolerance groups with weight loss. When the CDK-EPI_CysCr_ estimating equation was used, eGFR indexed to 1.73m^2^ BSA increased with weight loss. When the CKD-EPI_Cys_ estimating equation was used, eGFR indexed to 1.73m^2^ BSA increased with weight loss in the NFG and IFG groups but not in the T2DM group (Table 2). In contrast, eGFR indexed to actual BSA was consistently higher in subjects with IFG than NFG than T2DM (Table 2). eGFR indexed to actual BSA decreased significantly with weight loss within each glucose tolerance group and the proportion of patients with hyperfiltration decreased (Table 2).

Table 3 shows the characteristics of the 43 subjects (18 NFG, 13 IFG, and 12 T2DM) studied at baseline, 3-to-6 months, and 2-years. In general, there were modest increases in BMI, weight, BSA, and waist circumference between 3-to-6 months and 2-years. Fat-free mass was unchanged in the NFG and T2DM groups but decreased in the IFG group between 3-to-6 months and 2-years. In all three glucose tolerance groups, there was a trend towards increased fat mass between 3-to-6 months and 2-years. Median serum creatinine returned to baseline levels in the NFG, IFG, and T2DM groups at 2-years but cystatin C tended to decrease.

**Table 3:** Characteristics of subjects studied at baseline, 3-to-6 months, and 2-years. Data are presented as N(%),mean ± standard deviation, or median (interquartile range).

	Subjects Followed to 2-years			Glucose Tolerance Status								
Characteristic	N = 43			NFG N = 18			IFG N = 13			T2DM N = 12		
Age (years)	52 ± 7			49 ± 8			54 ± 4			54 ± 6		
Sex (% male)	60%			61%			69%			50%		
Race (% white)	93%			94%			92%			92%		
	**Baseline**	**3-to-6 Mos**	**2-years**	**Baseline**	**3-to-6 Mos**	**2-years**	**Baseline**	**3-to-6 Mos**	**2-years**	**Baseline**	**3-to-6 Mos**	**2-years**
BMI (kg/m^2^)	39 (37–42)	32 (29–33)§	33 (31–36)§	38 (36–39)	31 (29–32)§	33 (29–35)‡	39 (36–44)	32 (28–33)†	32 (31–35)†	40 (38–43)	33 (31–36)†	35 (33–37)†
Weight (kg)	117 (109–132)	94 (85–106)§	98 (89–110)§	116 (110–126)	91 (85–100)‡	98 (90–110)†	126 (113–148)	94 (89–114)*	100 (93–120)*	117 (104–126)	96 (86–105)*	96 (88–109)*
BSA (m^2^)	2.55 (2.44–2.75)	2.21 (2.08–2.39)§	2.28 (2.14–2.45)§	2.54 (2.45–2.68)	2.16 (2.07–2.30)‡	2.27 (2.15–2.45)‡	2.67 (2.49–2.97)	2.20 (2.13–2.51)*	2.30 (2.20–2.59)*	2.55 (2.36–2.68)	2.24 (2.09–2.37)*	2.25 (2.12–2.61)*
Waist circumference (cm)	121 (113–128)	101 (96–109)§	105 (98–118)§	98 (94–101)	98 (94–101)§	101 (97–112)†	102 (94–114)	102 (94–114)†	105 (102–121)*	106 (100–115)	106 (100–115)†	111 (102–120)*
Fat-free mass (kg)	65 (53–73)	64 (49–71)	62 (49–70)	68 (50–75)	66 (44–71)	65 (46–72)	66 (54–74)	66 (53–73)	61 (50–72)	63 (54–67)	57 (52–65)	60 (50–67)
Fat mass (kg)	52 (44–60)	33 (26–41)§	40 (32–46)§	50 (44–57)	30 (23–39)‡	37 (26–48)†	55 (41–60)	37 (26–41)*	38 (32–46)*	54 (47–61)	34 (31–42)†	41 (36–46)*
Systolic BP (mmHg)	130 (120–141)	120 (112–130) †	120 (111–135)*	129 (116–140)	124 (107–134)	119 (111–140)	137 (127–149)	120 (115–127)*	123 (119–134)	129 (120–134)	119 (109–127)	119 (108–132)
Serum Creatinine (mg/dl)	0.93 (0.84–1.05)	0.90 (0.83–1.01)	0.97 (0.87–1.05)	0.94 (0.84–1.05)	0.88 (0.83–0.95)	0.98 (0.89–1.04)	0.93 (0.84–1.08)	0.95 (0.88–1.05)	0.91 (0.87–1.09)	0.94 (0.87–1.02)	0.89 (0.80–1.00)	0.95 (0.88–1.02)
Cystatin C (mg/L)	0.80 (0.71–0.89)	0.80 (0.71–0.88)	0.74 (0.69–0.88)	0.79 (0.68–0.90)	0.73 (0.69–0.87)	0.73 (0.69–0.88)	0.79 (0.71–0.89)	0.80 (0.73–0.85)	0.74 (0.67–0.86)	0.82 (0.77–0.84)	0.84 (0.77–0.95)	0.83 (0.70–0.88)
U albumin-to-creatinine (mg/g)	4.3 (3.2–6.2)	5.0 (2.9–10.1)	4.0 (3.0–6.2)	4.6 (3.1–6.6)	4.8 (2.7–10.1)	4.3 (3.0–7.2)	3.7 (3.2–5.1)	5.2 (3.1–6.8)	3.6 (2.5–4.0)	4.8 (3.8–6.1)	6.0 (3.1–14.1)	4.3 (3.6–5.7)
UACR ≥30||	N = 2 (5%)	N = 1 (2%)	N = 2 (5%)	N = 1 (5%)	N = 0 (0%)	N = 1 (5%)	N = 0 (0%)	N = 0 (0%)	N = 0 (0%)	N = 1 (8%)	N = 1 (8%)	N = 1 (8%)

*p<0.05

†p<0.01

‡p<0.001

§p<0.0001 compared to baseline

|| cell size too small to do statistical testing

Table 4 shows eGFR indexed to 1.73m^2^ BSA and to actual BSA at baseline, 3-to-6 months, and 2-years by estimating equation in the subpopulation studied at 2-years. The effects seen in this subpopulation of 43 subjects at 3-to-6 months were qualitatively similar to those seen in the total population of 146 at 3-to-6 months. At 2-years, the MDRD and the CKD-EPI_Cr_, estimating equations indexed to 1.73m^2^ BSA showed a significant decrease in eGFR (p<0.01 and p<0.001 respectively) and the CKD-EPI_CysCr_ and CKD-EPI_Cys_ estimating equations indexed to 1.73m^2^ BSA showed no change in eGFR compared to 3-to-6 months. The CKD-EPI_Cr_ equation indexed to actual BSA showed an increase in eGFR at 2-years compared to 3-to-6 months (p<0.01). The MDRD, CKD-EPI_CysCr_, and CKD-EPI_Cys_ equations indexed to actual BSA showed no change between 3-to-6 months and 2-years. Changes in eGFR at 2-years by glucose tolerance group were qualitatively similar to those observed at 3-to-6 months. Between 3-to-6 months and 2-years, eGFR calculated with the CKD-EPI_Cr_, CKD-EPI_CysCr_ and CKD-EPI_Cys_ equations indexed to 1.73m^2^ BSA or to actual BSA generally remained unchanged across glucose tolerance groups (Table 4).

**Table 4:** eGFR indexed to 1.73m^2^ BSA and to actual BSA at baseline, 3-to-6 months and 2-years for subjects followed to 2-years by estimating equation and glucose tolerance status. Data are presented as N(%) or mean ± standard deviation.

	Subjects Followed to 2-years			Glucose Tolerance Status								
	N = 43			NFG N = 18			IFG N = 13			T2DM N = 12		
eGFR Equation	Baseline	3-to-6 Mos	2-year	Baseline	3-to-6 Mos	2-year	Baseline	3-to-6 Mos	2-year	Baseline	3-to-6 Mos	2-year
MDRD/1.73m^2^	77 ± 12	80 ± 11*	75 ± 9	77 ± 12	81 ± 10	75 ± 7	77 ± 13	78 ± 10	76 ± 12	74 ± 12	79 ± 14†	75 ± 11
≥135	0 (0%)	0 (0%)	0 (0%)	0 (0%)	0 (0%)	0 (0%)	0 (0%)	0 (0%)	0 (0%)	0 (0%)	0 (0%)	0 (0%)
120–134	0 (0%)	0 (0%)	0 (0%)	0 (0%)	0 (0%)	0 (0%)	0 (0%)	0 (0%)	0 (0%)	0 (0%)	0 (0%)	0 (0%)
90–119	7 (16%)	6 (14%)	3 (7%)	3 (17%)	3 (17%)	1 (6%)	3 (23%)	1 (8%)	1 (8%)	1 (8%)	2 (17%)	1 (8%)
60–89	34 (79%)	36 (84%)	38 (88%)	15 (83%)	15 (83%)	17 (94%)	9 (69%)	12 (92%)	11 (85%)	10 (83%)	9 (75%)	10 (83%)
<60	2 (5%)	1 (2%)	2 (5%)	0 (0%)	0 (0%)	0 (0%)	1 (8%)	0 (0%)	1 (8%)	1 (8%)	1 (8%)	1 (8%)
MDRD indexed to actual BSA	115 ± 27	104 ± 20§	101 ± 18§	114 ± 24	104 ± 19†	99 ± 13†	122 ± 33	104 ± 22†	105 ± 26†	110 ± 22	103 ± 21†	99 ± 17†
≥135	10 (23%)	2 (5%)	2 (5%)	3 (17%)	0 (0%)	0 (0%)	5 (38%)	1 (8%)	2 (15%)	2 (17%)	1 (8%)	0 (0%)
120–134	3 (7%)	8 (19%)	3 (7%)	2 (11%)	6 (33%)	2 (11%)	6 (46%)	1 (8%)	0 (0%)	1 (8%)	1 (8%)	1 (8%)
90–119	24 (56%)	22 (51%)	25 (58%)	12 (67%)	8 (44%)	11 (61%)	2 (15%)	8 (62%)	7 (54%)	6 (50%)	6 (50%)	7 (58%)
60–89	6 (14%)	11 (26%)	13 (30%)	1 (6%)	4 (22%)	5 (28%)	0 (0%)	3 (23%)	4 (31%)	3 (25%)	4 (33%)	4 (33%)
<60	0 (0%)	0 (0%)	0 (0%)	0 (0%)	0 (0%)	0 (0%)	0 (0%)	0 (0%)	0 (0%)	0 (0%)	0 (0%)	0 (0%)
CKD-EPI_Cr_/1.73m^2^	82 ± 11	82 ± 11§	81 ± 11§	84 ± 11	84 ± 11§	83 ± 11§	82 ± 11	82 ± 11§	81 ± 11§	81 ± 12	81 ± 12§	80 ± 11§
≥135	0 (0%)	0 (0%)	0 (0%)	0 (0%)	0 (0%)	0 (0%)	0 (0%)	0 (0%)	0 (0%)	0 (0%)	0 (0%)	0 (0%)
120–134	0 (0%)	0 (0%)	0 (0%)	0 (0%)	0 (0%)	0 (0%)	0 (0%)	0 (0%)	0 (0%)	0 (0%)	0 (0%)	0 (0%)
90–119	14 (33%)	14 (33%)	11 (26%)	6 (33%)	6 (33%)	6 (33%)	4 (31%)	4 (31%)	3 (23%)	4 (33%)	4 (33%)	2 (17%)
60–89	29 (67%)	29 (67%)	32 (74%)	12 (67%)	12 (67%)	12 (67%)	9 (69%)	9 (69%)	10 (77%)	8 (67%)	8 (67%)	10 (83%)
<60	0 (0%)	0 (0%)	0 (0%)	0 (0%)	0 (0%)	0 (0%)	0 (0%)	0 (0%)	0 (0%)	0 (0%)	0 (0%)	0 (0%)
CKD-EPI_Cr_ indexed to actual BSA	124 ± 24	106 ± 20§	109 ± 22§	124 ± 21	106 ± 19§	109 ± 21§	128 ± 29	108 ± 24§	111 ± 26§	119 ± 22	104 ± 18§	105 ± 18‡
≥135	12 (28%)	5 (12%)	7 (16%)	5 (28%)	2 (11%)	2 (11%)	5 (38%)	3 (23%)	4 (31%)	2 (17%)	0 (0%)	1 (8%)
120–134	6 (14%)	7 (16%)	5 (12%)	2 (11%)	3 (17%)	5 (28%)	0 (0%)	2 (15%)	0 (0%)	4 (33%)	2 (17%)	0 (0%)
90–119	23 (53%)	21 (49%)	22 (51%)	11 (61%)	9 (50%)	7 (39%)	7 (54%)	5 (38%)	7 (54%)	5 (42%)	7 (58%)	8 (67%)
60–89	2 (5%)	10 (23%)	9 (21%)	0 (0%)	4 (22%)	4 (22%)	1 (8%)	3 (23%)	2 (15%)	1 (8%)	3 (25%)	3 (25%)
<60	0 (0%)	0 (0%)	0 (0%)	0 (0%)	0 (0%)	0 (0%)	0 (0%)	0 (0%)	0 (0%)	0 (0%)	0 (0%)	0 (0%)
CKD-EPI_CysCr_/1.73m^2^	93 ± 14	95 ± 12	93 ± 12	95 ± 16	98 ± 13	94 ± 11	93 ± 11	94 ± 8	94 ± 13	90 ± 14	91 ± 13	92 ± 14
≥135	0 (0%)	0 (0%)	0 (0%)	0 (0%)	0 (0%)	0 (0%)	0 (0%)	0 (0%)	0 (0%)	0 (0%)	0 (0%)	0 (0%)
120–134	1 (3%)	1 (2%)	0 (0%)	1 (6%)	1 (6%)	0 (0%)	8 (62%)	0 (0%)	0 (0%)	0 (0%)	0 (0%)	0 (0%)
90–119	23 (53%)	27 (63%)	24 (56%)	9 (50%)	12 (67%)	11 (61%)	5 (38%)	9 (69%)	8 (62%)	6 (50%)	5 (50%)	5 (42%)
60–89	19 (44%)	15 (35%)	19 (44%)	8 (44%)	5 (27%)	7 (39%)	0 (0%)	4 (31%)	5 (38%)	6 (50%)	6 (50%)	7 (58%)
<60	0 (0%)	0 (0%)	0 (0%)	0 (0%)	0 (0%)	0 (0%)	0 (0%)	0 (0%)	0 (0%)	0 (0%)	0 (0%)	0 (0%)
CKD-EPI_CysCr_ indexed to actual BSA	139 ± 27	122 ± 19§	124 ± 20§	139 ± 26	124 ± 21§	123 ± 17‡	145 ± 27	124 ± 16†	128 ± 23†	132 ± 27	117 ± 19‡	121 ± 21*
≥135	20 (47%)	11 (26%)	13 (30%)	8 (44%)	6 (33%)	6 (33%)	7 (53%)	3 (23%)	4 (31%)	5 (42%)	2 (17%)	3 (25%)
120–134	13 (30%)	11 (26%)	8 (19%)	6 (33%)	4 (22%)	3 (17%)	5 (38%)	4 (31%)	3 (23%)	2 (17%)	3 (25%)	2 (17%)
90–119	10 (23%)	21 (49%)	21 (49%)	4 (22%)	8 (44%)	9 (50%)	1 (8%)	6 (46%)	5 (38%)	5 (42%)	7 (58%)	7 (58%)
60–89	0 (0%)	0 (0%)	1 (2%)	0 (0%)	0 (0%)	0 (0%)	0 (0%)	0 (0%)	1 (8%)	0 (0%)	0 (0%)	0 (0%)
<60	0 (0%)	0 (0%)	0 (0%)	0 (0%)	0 (0%)	0 (0%)	0 (0%)	0 (0%)	0 (0%)	0 (0%)	0 (0%)	0 (0%)
CKD-EPI_Cys_/1.73m^2^	100 ± 16	100 ± 15	102 ± 15	102 ± 19	103 ± 17	103 ± 17	101 ± 12	102 ± 10	103 ± 13	97 ± 16	95 ± 15	100 ± 14
≥135	0 (0%)	0 (0%)	0 (0%)	0 (0%)	0 (0%)	0 (0%)	0 (0%)	0 (0%)	0 (0%)	0 (0%)	0 (0%)	0 (0%)
120–134	2 (5%)	2 (5%)	4 (9%)	2 (11%)	2 (11%)	2 (11%)	0 (0%)	0 (0%)	1 (8%)	0 (0%)	0 (0%)	1 (8%)
90–119	31 (72%)	30 (70%)	31 (72%)	11 (61%)	12 (67%)	12 (67%)	10 (77%)	11 (85%)	11 (85%)	10 (83%)	7 (58%)	8 (67%)
60–89	10 (23%)	11 (26%)	8 (19%)	5 (28%)	4 (22%)	4 (22%)	3 (23%)	2 (15%)	1 (8%)	2 (17%)	5 (42%)	3 (25%)
<60	0 (0%)	0 (0%)	0 (0%)	0 (0%)	0 (0%)	0 (0%)	0 (0%)	0 (0%)	0 (0%)	0 (0%)	0 (0%)	0 (0%)
CKD-EPI_Cys_ indexed based to BSA	150 ± 28	129 ± 20§	136 ± 23§	150 ± 29	130 ± 23§	135 ± 24‡	156 ± 23	134 ± 15‡	140 ± 23†	143 ± 30	122 ± 21‡	132 ± 22*
≥135	29 (67%)	16 (37%)	20 (47%)	13 (73%)	8 (44%)	2 (11%)	10 (77%)	5 (38%)	7 (54%)	6 (50%)	3 (25%)	3 (25%)
120–134	9 (21%)	13 (30%)	13 (30%)	3 (17%)	5 (28%)	12 (66%)	2 (15%)	6 (46%)	5 (38%)	4 (33%)	2 (17%)	5 (42%)
90–119	5 (12%)	13 (30%)	9 (21%)	2 (11%)	4 (22%)	4 (22%)	1 (8%)	2 (15%)	1 (8%)	2 (17%)	7 (58%)	4 (33%)
60–89	0 (0%)	1 (2%)	1 (2%)	0 (0%)	1 (6%)	0 (0%)	0 (0%)	0 (0%)	0 (0%)	0 (0%)	0 (0%)	0 (0%)
<60	0 (0%)	0 (0%)	0 (0%)	0 (0%)	0 (0%)	0 (0%)	0 (0%)	0 (0%)	0 (0%)	0 (0%)	0 (0%)	0 (0%)

*p<0.05

†p<0.01

‡p<0.001

§p<0.0001 compared to baseline

## Discussion

In this study, we assessed the associations among demographic characteristics, measures of adiposity and body composition, serum creatinine, and cystatin C before and after substantial medical weight loss in individuals with NFG, IFG, and T2DM, and described the impact of severe obesity and weight loss on GFR estimated using creatinine-based and cystatin C-based equations indexed to 1.73m^2^ BSA and indexed to actual BSA. At baseline, serum creatinine was positively associated with male sex and fat-free mass, and negatively associated with BMI and fat mass. Cystatin C was positively associated with age, male sex, waist circumference, and diabetes but was not significantly associated with BMI, fat-free mass, or fat mass, confirming the results of a previous study that found no correlation between cystatin C, body weight, or fat free mass [8]. In multivariate analyses, change in serum creatinine at 3-to-6 months after weight loss was associated with measures of adiposity and body composition whereas cystatin C was not. This confirms that cystatin C may be preferable to serum creatinine when estimating renal function in the setting of substantial weight loss.

GFR estimating equations indexed to 1.73m^2^ BSA and based on serum creatinine alone resulted in lower estimates of GFR at baseline compared to estimating equations that incorporated cystatin C. Perhaps not surprisingly, GFR estimating equations indexed to actual BSA yielded substantially higher estimates of GFR at baseline and evidence of frequent renal hyperfiltration. Indexing eGFR to 1.73m^2^ BSA in severely obese participants failed to detect hyperfiltration and indeed suggested that between 92% and 27% had Stage 2 CKD.

Estimated GFR indexed to 1.73m^2^ BSA remained low but increased slightly after medical weight loss with greater increases occurring when GFR was estimated with the MDRD and CKD-EPI_CysCr_ equations. The mean eGFR did not change after weight loss when the CKD-EPI_Cr_ equation indexed to 1.73m^2^ BSA was used to estimate renal function, and increased slightly when the CKD-EPI_Cys_ equation was used. In general, the increases in eGFR were similar across groups by glucose tolerance status.

The results obtained when eGFR was indexed to actual BSA were in sharp contrast to those when GFR was indexed to 1.73m^2^ BSA. Estimated GFR calculated with the MDRD equation indexed to actual BSA remained lower than GFR calculated with the CKD-EPI_Cr_ equation, which remained lower than that calculated with the CKD-EPI_CysCr_ equation which, remained lower than that calculated with the CKD-EPI_Cys_ equation. Now, however, the mean eGFR indexed to actual BSA and calculated with each of the four estimating equations was consistently >100 ml/min and 15% to 61% of subjects exhibited hyperfiltration. In addition, with weight loss, eGFR indexed to actual BSA decreased, but on average remained ≥90 ml/min suggesting that weight loss ameliorates hyperfiltration in severely obese patients.

Recognizing the dramatic increase in the average BSA in the United States over the past 100 years, it is not unreasonable to index GFR to actual BSA in severely obese subjects. Previous studies in obese subjects have demonstrated that eGFR indexed to 1.73m^2^ BSA is substantially lower than measured GFR [21]. Chagnac and colleagues found that in 8 severely obese subjects, GFR measured directly using inulin was 145 ml/min before bariatric surgery and 110 ml/min after bariatric surgery. In a study by Friedman and colleagues, measured GFR decreased from 117 to 100 ml/min after bariatric surgery, whereas eGFR indexed to 1.73m^2^ BSA remained unchanged at 87 ml/min/1.73m^2^ before and after bariatric surgery [22]. These results are quite consistent with our findings using GFR indexed to actual BSA before and after substantial medical weight loss. It has been previously reported that when GFR is indexed to 1.73m^2^ BSA, the reduction in glomerular hyperfiltration after bariatric surgery is “masked” [22].

In summary, we have demonstrated that in severely obese subjects, creatinine-based GFR estimating equations indexed to 1.73m^2^ BSA substantially underestimate renal function relative to cystatin C-based GFR estimating equations. This is more of a problem with the MDRD equation than with the CKD-EPI_Cr_ equation. The MDRD equation indexed to 1.73m^2^ BSA results in a substantially higher prevalence of Stage 2 CKD among severely obese subjects without objective evidence of kidney disease. In contrast, both the CKD-EPI_CysCr_ and the CKD-EPI_Cys_ equations indexed to 1.73m^2^ BSA result in higher estimates of GFR among severely obese subjects. All four equations when indexed to 1.73m^2^ BSA fail to detect renal hyperfiltration. Estimating GFR based on actual BSA results in substantially higher GFR estimates and suggests that severely obese subjects with NFG, IFG, and T2DM exhibit renal hyperfiltration which is reduced following medical weight loss at both 3-to-6 months and 2-years.

The major limitation of our study is that we did not have a direct measure of GFR in any of the subjects. Nevertheless, our results indicate that GFR estimating equations indexed to 1.73m^2^ BSA result in lower estimates of GFR especially when creatinine-based estimating equations are used. Using cystatin C-based estimating equations and indexing GFR to actual BSA gives GFR estimates that are more consistent with measured GFR levels observed in other studies of severely obese patients before and after weight loss [18]. Our results following medical weight loss suggest that renal hyperfiltration is ameliorated at both 3-to-6 months and at 2-years and that renal function remains stable. Taken together, these results suggest that substantial medical weight loss in people with severe obesity may reduce renal risk.
